# Chemical Composition, Nutritional, and Biological Properties of Extracts Obtained with Different Techniques from *Aronia melanocarpa* Berries

**DOI:** 10.3390/molecules29112577

**Published:** 2024-05-30

**Authors:** Alessandra Piras, Silvia Porcedda, Antonella Smeriglio, Domenico Trombetta, Mariella Nieddu, Franca Piras, Valeria Sogos, Antonella Rosa

**Affiliations:** 1 Department of Chemical and Geological Sciences, University of Cagliari, Cittadella Universitaria, SP 8, Monserrato-Sestu km 0.700, 09042 Monserrato, CA, Italy; porcedda@unica.it; 2 Department of Chemical, Biological, Pharmaceutical and Enviromental Sciences, University of Messina, Viale Ferdinando Stagno d’Alcontres 31, 98166 Messina, SI, Italy; antonella.smeriglio@unime.it (A.S.); domenico.trombetta@unime.it (D.T.); 3 Department of Biomedical Sciences, University of Cagliari, Cittadella Universitaria, SS 554, km 4.5, 09042 Monserrato, CA, Italy; mnieddu@unica.it (M.N.); sogos@unica.it (V.S.)

**Keywords:** *Aronia melanocarpa*, natural extracts, berries, essential oil, fixed oil, phenolic extract

## Abstract

This study investigates the chemical composition, nutritional, and biological properties of extracts obtained from *A. melanocarpa* berries using different extraction methods and solvents. Hydrodistillation and supercritical fluid extraction with CO_2_ allowed us to isolate fruit essential oil (HD_EX_) and fixed oil (SFE_EX_), respectively. A phenol-enriched extract was obtained using a mild ultrasound-assisted maceration with methanol (UAM_M_). The HD_EX_ most abundant component, using gas chromatography-mass spectrometry (GC/MS), was italicene epoxide (17.2%), followed by hexadecanoic acid (12.4%), khusinol (10.5%), limonene (9.7%), dodecanoic acid (9.7%), and (E)-anethole (6.1%). Linoleic (348.9 mg/g of extract, 70.5%), oleic (88.9 mg/g, 17.9%), and palmitic (40.8 mg/g, 8.2%) acids, followed by α-linolenic and stearic acids, were the main fatty acids in SFE_EX_ determined using high-performance liquid chromatography coupled with a photodiode array detector and an evaporative light scattering detector (HPLC-DAD/ELSD). HPLC-DAD analyses of SFE_EX_ identified β-carotene as the main carotenoid (1.7 mg/g), while HPLC with fluorescence detection (FLU) evidenced α-tocopherol (1.2 mg/g) as the most abundant tocopherol isoform in SFE_EX_. Liquid chromatography-electrospray ionization-MS (LC-ESI-MS) analysis of UAM_M_ showed the presence of quercetin-sulfate (15.6%, major component), malvidin 3-*O*-(6-*O*-p-coumaroyl) glucoside-4-vinylphenol adduct (pigment B) (9.3%), di-caffeoyl coumaroyl spermidine (7.6%), methyl-epigallocatechin (5.68%), and phloretin (4.1%), while flavonoids (70.5%) and phenolic acids (23.9%) emerged as the most abundant polyphenol classes. UAM_M_ exerted a complete inhibition of the cholesterol oxidative degradation at 140 °C from 75 μg of extract, showing 50% protection at 30.6 μg (IA_50_). Furthermore, UAM_M_ significantly reduced viability (31–48%) in A375 melanoma cells in the range of 500–2000 μg/mL after 96 h of incubation (MTT assay), with a low toxic effect in normal HaCaT keratinocytes. The results of this research extend the knowledge of the nutritional and biological properties of *A. melanocarpa* berries, providing useful information on specific extracts for potential food, cosmetic, and pharmaceutical applications.

## 1. Introduction

The use of medicinal herbs /plants and their isolated extracts/compounds in the prevention and treatment of various diseases is an ancient practice, and the modern drug discovery process is greatly derived from herbal traditional medicine [1]. The identification of bioactive compounds from natural sources and the evaluation of their potential use in health promotion are constantly attempted [1]. Phenolic compounds (phenolic acids, flavonoids, stilbenes, coumarins, and lignans) from herbs/plants have attracted increased attention for their various biological activities [2]. Moreover, there is growing interest in the lipid characteristics of non-conventional vegetable oils as a source of bioactive constituents and functional nutrients (phytochemicals, fat-soluble vitamins, and essential fatty acids) for human nutrition and medical applications [3,4].

*Aronia melanocarpa* (Michx.) Elliot, belonging to the Rosaceae family, is a plant known as “Black Aronia” based on the color of its berries (black chokeberry) [5,6]. The *A. melanocarpa* plant is native to the eastern parts of North America [5,6,7,8,9]. In the 1900s, it was introduced to some European countries (Russia, Poland, Germany, Sweden, Norway, and Finland, among others) and in 1976 to Japan [5,6,7,8]. Now the plant is cultivated almost all over the world, but it has gained the highest popularity in Europe where it is cultivated as an important industrial crop, providing fruits used by the food industry [5,6,8].

Native Americans used *A. melanocarpa* berries for food and medicinal application, in particular as an infusion to treat colds, while the bark was used as an astringent [5,8,9]. The *A. melanocarpa* edible black berries (diameter of about 1 cm), characterized by a little sour and astringent flavor [5,8], are used in the food industry for the production of syrups, juices, jams, and jellies (thanks to the presence of pectin), preserves, purees, nectars, wines, schnapps, tinctures, fruit infusions (tea), baked products, fruit desserts, and food supplements [5,6,8,9,10,11]. The harvest of chokeberries is generally performed during August and September [5,8,11].

*A. melanocarpa* berries are widely recognized as a valuable source of bioactive phenolic compounds such as flavonoids (mainly anthocyanins, proanthocyanidins, flavanols, and flavonols), phenolic acids (chlorogenic, neochlorogenic, caffeic), and tannins [5,6,7,8,9,10,11,12]. Polyphenolic compounds represent the most important constituents of black *A. melanocarpa* berries, accounting for 10.6–197.0 mg/g dry weight (dw) and 6.9–25.6 mg/g fresh weight (fw) as gallic acid equivalents [5,8], and the water-soluble pigment anthocyanins are responsible for the characteristic chokeberry black and dark blue/violet color [9,11].

Moreover, carbohydrates (sorbitol, fructose, glucose, and sucrose), organic acids (quinic, malic, citric, shikimic, oxalic, and succinic acids), amino acids, proteins, minerals (K, Ca, P, Mg, Na, Fe, Se, and Zn), vitamins (ascorbic acid, vitamins E, B, and K), aromatic compounds, fibers, fatty acids, carotenoids, and essential oil have been identified among berry components [5,6,7,8,9,10,11,13]. Values from 0.09% to 0.17% have been reported for the total lipid content in fresh chokeberry fruits, mostly represented by glycerides (linoleic acid as the main fatty acid), followed by phospholipids and sterols (mainly β-sitosterol) [11,13]. Among the volatile constituents of black chokeberry, benzaldehyde cyanohydrin, hydrocyanic acid, and benzaldehyde have been identified as the main compounds [13].

The chemical composition of *A. melanocarpa* berries depends on several factors, including environmental and climatic conditions (the area of cultivation and the soil characteristics), cultivation techniques, genetic attributes (cultivar, genotype, and variety), maturity of the berries, hydration and harvesting methods, and storage conditions [5,6,8,9,11].

Extracts and compounds obtained from *A. melanocarpa* berries are widely used in the food, cosmetic, and pharmaceutical sectors for their positive health effects, including antioxidant [5,6,7,8,9,11,13,14], anti-inflammatory [5,8,9,14,15], anticancer [5,8,9,13,14], anti-aging [5], antimicrobial [5,7,8,14], antiviral [8,9,14], antidiabetic [5,8,9,13,14], anti-atherosclerotic [8,9], anti-adipogenic [12], lipid-lowering [9,16], antimutagenic [5,8,9,13], hypotensive [5,8,9,13], hepatoprotective [8,9,13], gastroprotective [8,9], neuroprotective [14], and cardioprotective [13] properties.

Different polar solvents/extraction processes have been used to obtain phenol-enriched extracts from the *A. melanocarpa* fresh/dried berries, such as shaking at room temperature with 80% acetone/0.5% formic acid [6], maceration with ethanol [7] or 50–99% ethanol [8,16,17], extraction with 70% ethanol in ultrasonic water bath [18], decoction, and infusion [8,18]. n-Hexane in a Soxhlet apparatus was used for fixed-oil extraction from *A. melanocarpa* dried berries [19], whereas fruit volatiles were obtained using a hydraulic press [20] with simultaneous distillation and extraction (SDE) and solvent techniques [21].

The main objective of this work was to study the chemical composition, nutritional, and biological properties of several extracts obtained from the berries of *A. melanocarpa* using different extraction methods and solvents.

Hydrodistillation (HD) in a Clevenger-type apparatus [22] was used to obtain the essential oil (HD_EX_) from black chokeberries. Its volatile component profile was determined using gas chromatography-mass spectrometry (GC-MS).

Fixed oil from *A. melanocarpa* berries (SFE_EX_) was obtained using supercritical fluid extraction with carbon dioxide (SFE-CO_2_) [3,22]. Non-polar solvent extraction in a Soxhlet apparatus represents the most employed method for extracting oils from vegetable matrices. However, the use of supercritical CO_2_ for oil extraction has several advantages in the food industry, leading to less deterioration of the thermally labile components and the absence of toxic residual solvents in the final products [22,23,24]. SFE_EX_ was characterized for fatty acid (FA) profile using a high-performance liquid chromatography system coupled with a photodiode array detector and evaporative light scattering detector (HPLC-DAD/ELSD). The quali-quantitative analysis of carotenoids and tocopherols in SFE_EX_ was carried out using HPLC-DAD and HPLC coupled to fluorescence detection (FLU), respectively. A mild ultrasound-assisted maceration (UAM) [25,26] with the conventional solvent n-hexane was also used to obtain a fixed oil (UAM_H_) from *A. melanocarpa* berries for FA comparison with SFE_EX_.

Finally, UAM with methanol was applied to obtain a phenol-enriched extract (UAM_M_) from black chokeberries. The UAM_M_ phytochemical profile was obtained using liquid chromatography-electrospray ionization-MS analysis (LC-ESI-MS). Moreover, the UAM_M_’s effect on cell viability (96 h of incubation) was tested in human skin melanoma A375 cells [27,28] and human skin HaCaT keratinocytes, together with the investigation of its ability to preserve the thermal (140 °C) solvent-free oxidation of cholesterol. An in vitro model of lipid oxidation was amply used to assess the antioxidant properties of natural extracts and compounds [29].

## 2. Results

### 2.1. Preparation of Extracts from A. melanocarpa Berries and Extraction Yields

**Scheme 1:**
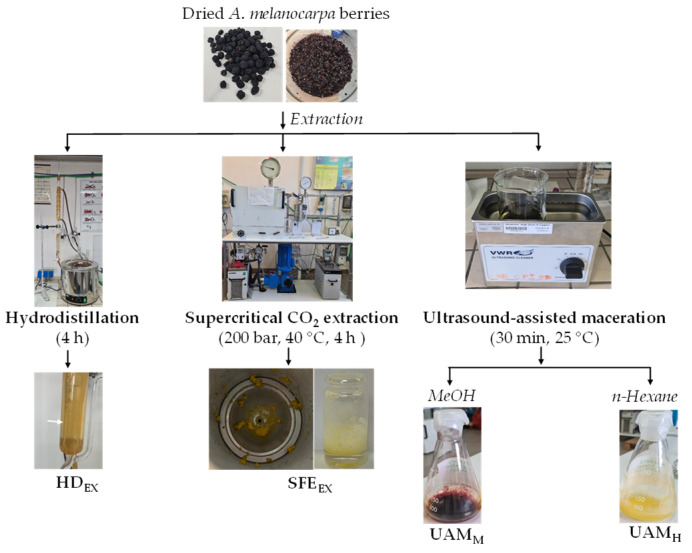
Scheme of preparation of HD_EX_, SFE_EX_, UAM_M_, and UAM_H_ extracts obtained from dried berries of *A. melanocarpa* using hydrodistillation in a Clevenger-type apparatus, supercritical CO_2_ extraction, and ultrasound-assisted maceration with MeOH and n-hexane, respectively.

Four different extracts (HD_EX_, SFE_EX_, UAM_M_, and UAM_H_) were obtained from dried berries of *A. melanocarpa* with several extraction procedures, as reported in Scheme 1.

The hydrodistillation (4 h) of ground portions of *A. melanocarpa* berries resulted in the extraction of the volatile fraction (HD_EX_) with an unquantifiable yield, as the pale-yellow oil remained attached to the walls of the Clevenger apparatus and was recovered by washing with n-hexane.

Fixed oil (SFE_EX_) was obtained from dried *A. melanocarpa* fruits using CO_2_-SFE extraction at higher pressure (200 bar) and 40 °C (ρCO_2_ = 0.840 g cm^3^) for 4 h with a yield of 0.2% *w*/*w*. We have previously used these CO_2_-SFE operating parameters for the extraction of fixed oils from different vegetable matrices [3,22]. The yield of *A. melanocarpa* oil extract (UAM_H_) obtained using UAM for 30 min with n-hexane was 0.4% *w*/*w*.

The yield of the phenol-enriched extract (UAM_M_), obtained with UAM for 30 min at 25 °C with MeOH, was 29% *w*/*w* of the dry weight.

### 2.2. Essential Oil Composition

**Figure 1:**
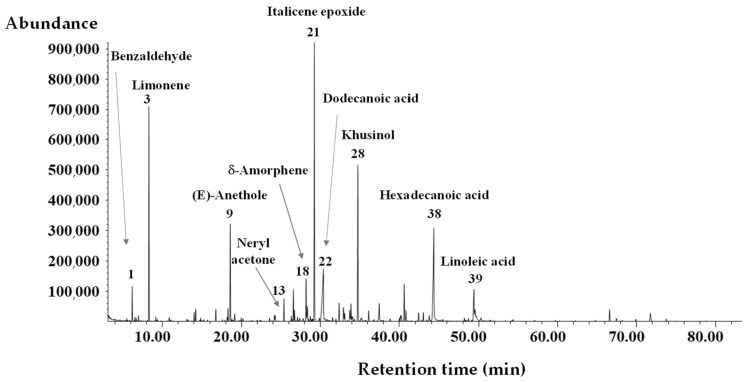
GC-MS chromatogram, obtained on an HP-5ms capillary column, of *A. melanocarpa* fruit essential oil (HD_EX_), with the indication of the main volatile compounds as reported in Table 1.

The HD_EX_ chemical analyses were focused on both qualitative and quantitative data. The GC-MS technique was used to reveal the chemical composition. Figure 1 shows the chromatographic profile using GC–MS technique, with the indication of the main identified volatile compounds and the chemical composition, expressed as % area, reported in Table 1.

GC-MS analysis revealed the presence of 42 volatile compounds, and among them, 28 components were identified, representing 86.7% of the total organic volatiles.

HD_EX_ was characterized by a prevalence of oxygenated sesquiterpenes constituting the major class with 30.9%, together with the class that includes compounds of various kinds, of which mainly fatty acids (30.9%), accompanied by noticeable contents of hydrocarbons and oxygenated monoterpenes (9.9% and 9.5%, respectively) and much smaller amounts of hydrocarbons sesquiterpenes (5.5%). Italicene epoxide was found to be the major component, accounting for 17.2% of total compounds, followed by hexadecanoic acid (12.4%), khusinol (10.5%), limonene (9.7%), dodecanoic acid (9.7%), and (E)-anethole (6.1%). Other components with relatively small amounts were linoleic acid (3.1%), δ-amorphene (2.9%), benzaldehyde (1.6%), neryl acetone (1.4%), 1,10-di-epi-cubenol (1.3%), and γ-cadinene (1.3%).

### 2.3. Fatty Acid Profile of Oil Extracts (SFE_EX_ and UAM_H_)

**Figure 2:**
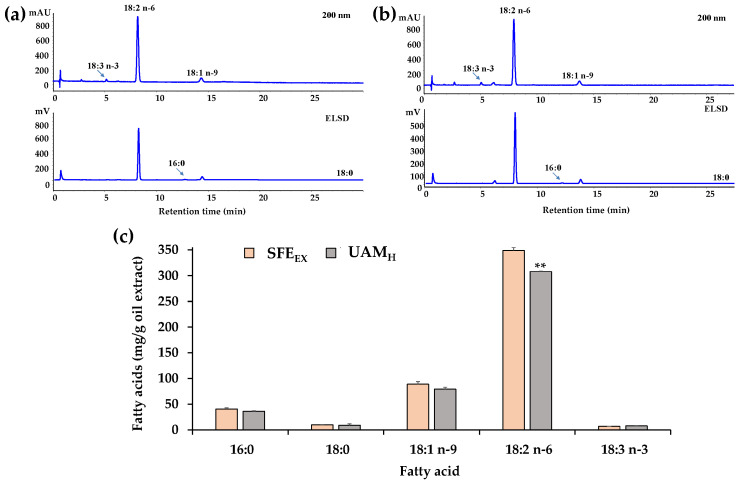
Fatty acid (FA) chromatographic profiles (HPLC analysis) obtained using DAD (200 nm, unsaturated FA) and ELSD (saturated FA) detection of *A. melanocarpa* fruit oil extracts SFE_EX_ (**a**) and UAM_H_ (**b**). FA composition (expressed as mg/g of oil extract) determined using HPLC-DAD/ELSD analysis of *A. melanocarpa* SFE_EX_ and UAM_H_ oil extracts after saponification (**c**). Data are expressed as mean values ± standard deviations (SD) (*n* = 4); ** = *p* < 0.01 versus SFE_EX_ (Student’s unpaired *t*-test with Welch’s correction).

The chromatographic FA profile determined using HPLC-DAD/ELSD analysis of oil extract SFE_EX_ obtained from *A. melanocarpa* fruits using CO_2_-SFE extraction is reported in Figure 2a.

Figure 2b displays the chromatographic FA profile of UAM_H_ obtained, for comparison, from *A. melanocarpa* fruits using UAM with n-hexane, the conventional lipophilic solvent normally used for oil extraction.

Figure 2c shows the FA composition (expressed as mg/g of oil extract) determined by HPLC-DAD/ELSD analysis of *A. melanocarpa* oil extract (SFE_EX_) after mild saponification. The combined use of the two detectors allowed us to identify and quantify both saturated FA (SFA, ELSD detection) and unsaturated FA (UFA, UV detection at a wavelength of 200 nm) in one single analysis [33]. SFE_EX_ showed a concentration of approximately 10.3% saturated FA (SFA), mainly palmitic acid 16:0 (8.2%, 40.8 ± 2.3 mg/g of extract) and stearic acid 18:0 (2.0%, 10.0 ± 0.5 mg/g of extract), 17.9% of oleic acid 18:1 n-9 (88.9 ± 5.1 mg/g of extract), and 71.8% of polyunsaturated FA (PUFA), mainly constituted by the essential FA linoleic acid (18:2 n-6) and α-linolenic acid (18:3 n-3), which represented 70.4% (348.9 ± 5.8 mg/g of extract) and 1.4% (6.8 ± 0.3 mg/g of extract) of the FA content, respectively.

Moreover, the oxidative status of SFE_EX_ was evaluated using the HPLC determination of the level of conjugated diene FA hydroperoxides (HP). The oil showed an average HP content of 21.6 ± 1.5 μmol/g of SFE_EX_.

The FA composition of SFE_EX_ was similar to that of UAM_H_ oil extract obtained with hexane extraction (Figure 2c). The most represented fatty acids of UAM_H_ extract were 18:2 n-6 (307.8 ± 0.8 mg/g of extract, 69.9%), 18:1 n-9 (79.3 ± 3.6 mg/g of extract, 18%), and 16:0 (36.4 ± 0.5 mg/g of extract, 8.3%), and, in particular, the essential fatty acid 18:3 n-3 averaged 7.8 mg/g ± 0.6 mg/g of extract. A significant difference (*p* < 0.01) was determined between SFE_EX_ and UAM_H_ only for the linoleic acid amount, while similar amounts were measured for other FA.

### 2.4. Quali-Quantitative Determination of Carotenoids and Tocopherols in SFE_EX_

**Figure 3:**
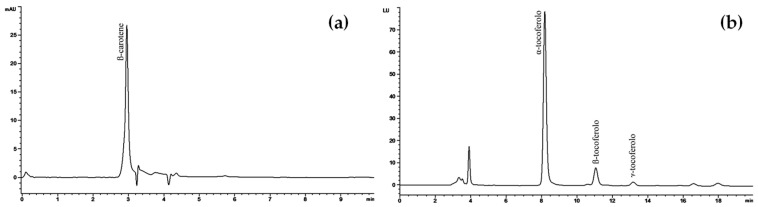
Quali-quantitative analysis of SFE_EX_ carotenoids (**a**) and tocopherols (**b**) using HPLC-DAD (474 nm) and HPLC-FLU (λ_ex_ 295 nm, λ_em_ 330 nm) analysis.

Then, HPLC-DAD and HPLC-FLU analyses allowed us to identify and quantify the carotenoid and tocopherol content of the SFE_EX_ oil extract, as depicted in Figure 3a and Figure 3b, respectively.

SFE_EX_ showed β-carotene as the main carotenoid (170.16 ± 8.55 mg/100 g of extract). Among tocopherols, only the three isoforms α, β, and γ were detected. α-Tocopherol represented the most abundant isoform (125.31 ± 2.65 mg/100 g of extract), followed by β-tocopherol (17.07 ± 0.44 mg/100 g) and γ-tocopherol (4.36 ± 0.05 mg/100 g).

### 2.5. Analysis of Polyphenols in UAM_M_

The phytochemical profile of UAM_M,_ obtained using ultrasound-assisted maceration with methanol, was tentatively elucidated using LC-ESI-MS analysis. The results, expressed as mean area percentage (%) ± standard deviation (*n* = 3) of each compound in comparison to the total area of polyphenols identified, are shown in Table 2.

Fifty-seven compounds were identified. Flavonoids were the most abundant class of polyphenols identified (70.47%), followed by phenolic acids (23.94%) and other minor compounds (5.60%). Among flavonoids, flavonols represented the most abundant sub-class (22.09%), followed by anthocyanins (19.99%), flavanones (10.02%), flavones (9.34%), and flavanols (9.03%). Quercetin-sulfate (15.58%) emerged as the major component, followed by malvidin 3-*O*-(6-*O*-p-coumaroyl) glucoside-4-vinylphenol adduct (pigment B) (9.28%), di-caffeoyl coumaroyl spermidine (7.60%), methyl-epigallocatechin (5.68%), and phloretin (4.09%).

### 2.6. Protective Effect of UAM_M_ against Cholesterol Degradation

UAM_M_ was assayed for its antioxidant activity during cholesterol oxidation. The consumption of cholesterol in a dry state at 1 h oxidation at 140 °C, and the formation of its major oxidation products, 7-ketocholesterol (7-keto) and 7β-hydroxycholesterol (7β-OH), were measured as markers of the oxidative process.

**Figure 4:**
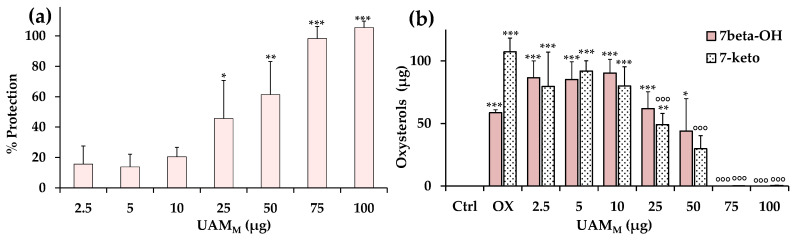
(**a**) Antioxidant activity (% protection) of different amounts (2.5–100 μg) of *A. melanocarpa* UAM_M_ berry extract measured during cholesterol oxidation at 140 °C for 1 h. Three independent experiments were performed, and data are presented as mean and standard deviation (*n* = 3); *** = *p* < 0.001, ** = *p* < 0.01, * = *p* < 0.05 versus oxidized controls (0% protection). (**b**) Values of oxysterols 7β-OH and 7-keto (expressed as μg) measured in the controls (Ctrl) and in oxidized samples in the absence (0) or in the presence of different amounts (2.5–100 μg) of UAM_M_ during cholesterol oxidation; data are presented as mean ± SD (*n* = 3). *** = *p* < 0.001, ** = *p* < 0.01, * = *p* < 0.05 versus Ctrl; °°° = *p* < 0.001, versus oxidized controls (0). Statistical analyses are performed with one-way ANOVA and Bonferroni post test.

Figure 4 shows the antioxidant activity measured during cholesterol oxidation in the presence of different amounts (2.5–100 μg) of UAM_M_.

At 140 °C, cholesterol was an oil, and more than 80% of the initial compound disappeared within 1 h heating, coupled with an increase in the main stable oxidative products 7-keto and 7β-OH, as previously observed [29]. Antioxidant activity is reported as the percentage of cholesterol protection, calculated considering the percent of sterol consumption in the presence of the UAM_M_ with respect to total cholesterol consumption without UAM_M_ (100% of consumption or 0% of protection). UAM_M_ exerted a significant protection (45%) of cholesterol from degradation at 25 μg, showing complete inhibition of the oxidative process from 75 μg, with an IA_50_ value (amount of antioxidant that gives a 50% protection) of 30.6 μg (Figure 4a).

Figure 4b shows the values (expressed as μg) of the oxysterols 7β-OH and 7-keto measured during cholesterol oxidation in the controls (Ctrl) and in samples oxidized in the absence (oxidized samples, 0) or in the presence of different amounts (2.5–100 μg) of UAM_M_. In our experimental conditions, 7β-OH was formed in a greater amount in the first part of the cholesterol oxidative process (30 min), while after 1 h oxidation, 7-keto represented the most abundant stable degradative product, also derived from 7β-OH oxidation. UAM_M_ significantly reduced, in comparison to oxidized samples, the formation of 7-keto from 25 μg, preventing the formation of both oxysterols from 75 μg, confirming the inhibition of the cholesterol oxidative degradation.

### 2.7. Cytotoxic Effect of UAM_M_ in Cancer A375 Cells and HaCaT Keratinocytes

Finally, the cytotoxicity of UAM_M_ was investigated using the MTT viability colorimetric assay in human diploid immortalized skin malignant melanoma (A375) cells, a cultured cancer cell model amply used to assess the cytotoxic effect and potential antitumor properties of compounds/extracts from natural sources in cutaneous melanoma [27,28]. Moreover, the UAM_M_’s effect on cell viability was also monitored in human HaCaT keratinocytes as normal skin cell line.

**Figure 5:**
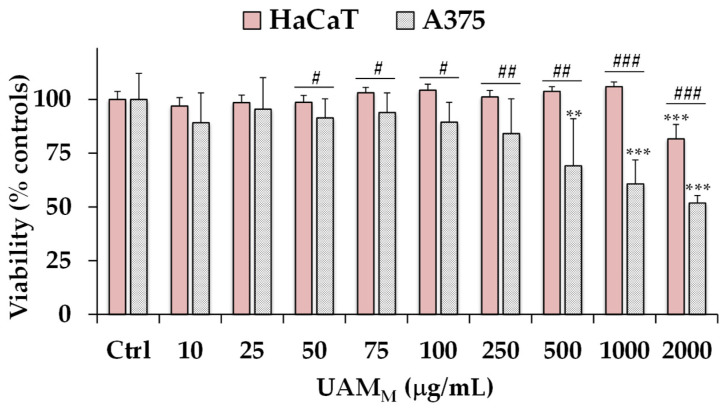
Viability, expressed as % of the control (Ctrl), induced using incubation for 96 h with different amounts (10–2000 μg/mL) of *A. melanocarpa* UAM_M_ berry extract in A375 human melanoma cells and human HaCaT keratinocytes (MTT assay). Data are presented as mean and standard deviation (*n* = 12). *** = *p* < 0.001, ** = *p* < 0.01 versus respective Ctrl (statistical analyses were performed using one-way ANOVA and Bonferroni post test). For each concentration group: ^###^ = *p* < 0.001, ^##^ = *p* < 0.01, ^#^ = *p* < 0.05, for A375 cells versus HaCaT cells (Student’s unpaired *t*-test with Welch’s correction).

Figure 5 shows the viability, expressed as % of control cells, induced by 96 h-incubation with different amounts (from 10 to 2000 μg/mL) of *A. melanocarpa* UAM_M_ berry extract in A375 melanoma cells and HaCaT keratinocytes using an MTT assay.

A dose-dependent growth inhibition was observed in treated cancer A375 cells, in comparison with control (untreated) cells, from the concentration of 250 μg/mL. UAM_M_ exerted a significant viability reduction, versus controls, from 31 to 48% at the dose range of 500–2000 μg/mL.

No marked changes in cell viability, in comparison to control cells, were observed in HaCaT cells treated with UAM_M_ in the range 10–1000 μg/mL, while a 18% viability reduction was observed at 2000 μg/mL, indicating a more selective toxicity towards malignant cells than normal cells.

## 3. Discussion

*A. melanocarpa* berries (black chokeberry) are a rich source of many bioactive components, with a wide range of health-promoting properties [5,8,9,11,13]. The chemical composition and biological properties of *A. melanocarpa* berry extracts have been demonstrated to greatly depend on the solvent and extraction method used that affect the type and concentration of components (based on polarity, volatility, and degradability) [6].

There is a great interest in non-conventional vegetable oils (essential and fixed oils) from medicinal plants as a source of bioactive constituents and functional nutrients (phytochemicals, essential fatty acids, and fat-soluble vitamins) for nutritive as well as medicinal purposes [3,4,33,34]. In this work, essential oil (HD_EX_) and fixed oil (SFE_EX_ and UAM_H_) were obtained from dried whole berries of *A. melanocarpa* using different extraction methods and were characterized for their main chemical components.

Hydrodistillation in a Clevenger-type apparatus was used to obtain the fruit essential oil HD_EX_. In the current literature, some manuscripts reported the extraction and characterization of *A. melanocarpa* volatile compounds [13,20,21,35,36]. However, to the best of our knowledge, no previous study reported the volatile fraction obtained with HD. HD_EX_ was extracted from dried *A. melanocarpa* berries with a low yield, and GC-MS analysis revealed the prevalence of oxygenated sesquiterpenes among volatiles, and italicene epoxide was identified as the major component, followed by khusinol, limonene, and (E)-anethole. Low amounts of δ-amorphene, benzaldehyde, neryl acetone, 1,10-di-epi-cubenol, and γ-cadinene were also detected. Several free fatty acids, such as dodecanoic acid (12:0), hexadecanoic acid, and linoleic acid, were also identified in HD_EX_.

Our results differed from those previously reported for the composition of volatiles extracted from *A. melanocarpa* berries with different methods [13,20,21,35,36], mainly due to the different applied extraction techniques.

GC-MS and GC with flame ionization detector (GC-FID) analyses of volatile constituents obtained from *A. melanocarpa* berries using solid-phase microextraction (SPME) evidenced the presence of benzaldehyde, hexanal, trans-2-hexenal, cis-3-hexenol, ethanol, hexan-1-ol, and ethyl-ethanoate as the most abundant compounds, highlighting considerable variation in the berry volatile profiles related to climatic conditions, cultivar, habitat, and time of collection [35].

The GC-MS analysis of the extract obtained by redistillation, with diethyl etherpentane in a Kutscher-Steudel extraction apparatus, of the juice obtained by pressing deep-frozen black chokeberries in a hydraulic press, evidenced benzaldehyde cyanohydrin (2.8 mg/kg), hydrocyanic acid (1.1 mg/kg), and benzaldehyde (0.21 mg/kg) as the main volatile constituents [13,20]. In addition, a series of benzene derivatives like benzyl alcohol, 2-phenylethanol, phenylacetaldehyde, salicylaldehyde, acetophenone, 2-hydroxy acetophenone, 4-methoxyacetophenone, phenol, 2-methoxy phenol, and methyl benzoate have also been reported as aroma components, while terpene hydrocarbons were identified in trace amounts [13,20].

The analysis of the profile of volatile constituents of *A. melanocarpa* berries from two different genotypes, extracted using headspace-solid-phase microextraction (HS-SPME) and simultaneous distillation/extraction (SDE), evidenced 3-penten-2-one, 3,9-epoxy-p-menth-1-ene, and benzaldehyde as the most abundant constituents. However, their percentage concentrations were remarkably different in the HS-SPME and SDE profiles [21]. SDE extracted a higher number of terpenoids than the SPME, while the SPME extracts contained more alcohols and esters; moreover, the majority of the identified volatiles in the juice HS were most likely derived from enzymatic degradation of fatty acids and amino acids [21]. Few volatile compounds were measured with GC-MS analysis in the extract obtained using SPME extraction from crushed chokeberry dried fruits from a Croatian market, and 3-penten-2-one was the most abundant compound [36].

Hydrodistillation of whole dried *A. melanocarpa* berries led to an essential oil (HD_EX_) characterized by a high content of oxygenated sesquiterpenes, and among volatile compounds, a conspicuous amount of free FA was also detected. Previous studies evidenced the presence of FA in the essential oil extracted using HD from *Helichrysum italicum* stems (with leaves and flower heads) in a Clevenger-type apparatus [37]. Essential oils of many plants contain esters derived from the combinations of alcohols with FA (volatiles or semi-volatile compounds), and the presence of free FA, could be attributable to hydrolytic processes under HD conditions [37].

In this work, SFE-CO_2_ extraction was applied to obtain oil extract (SFE_EX_) from whole dried *A. melanocarpa* berries. Novel and greener methods, such as microwaves, ultrasound-assisted extraction, and supercritical fluid extraction, have been amply used in recent years to obtain fixed oils from vegetable matrices [22,23,24]. Specifically, SFE-CO_2_ extraction is an alternative, environmentally benign, separation technique, amply used to obtain fixed oils from spices/plants [3,22,33]. Few studies previously reported the FA composition of oil extract obtained from *A. melanocarpa* berries [9,13,19,38,39], particularly with SFE-CO_2_ extraction [39].

SFE**_EX_**, analyzed using liquid chromatographic methods, showed a composition characterized by a high ratio of UFA (approximately 90% of total FA) to SFA. A high content of PUFA was determined in SFE**_EX_**, mainly constituted by the essential FA linoleic acid and α-linolenic acid (total value of 356 mg/g of extract), compounds indispensable for human health that cannot be synthesized de novo by humans and must be obtained from dietary intake [40]. Interestingly, the FA composition of SFE**_EX_** was quite similar to that of UAM_H_, obtained using UAM with n-hexane, the conventional solvent used for oil extraction from vegetable matrices [22,24].

The FA profile of *A. melanocarpa* fruit oil was comparable to that previously reported in the literature. The seeds of *A. melanocarpa* berries, extracted in a Soxhlet apparatus with hexane for 8 h, have been reported to contain glyceride oil (19.3 g/kg fw) and phospholipids (2.8 g/kg fw) [19]. The total fat content of berries was reported to be 0.14 g/100 g fw [9,13], while high fat content (3–14%) was reported in dried pomace of chokeberries [9,38]. Linoleic acid, followed by oleic and palmitic acids, have been identified as the main FA in chokeberry seed oils [13,19]. A high content of UFA (90.5%) was reported in the dried pomace of chokeberries, characterized by linoleic acid as the most abundant FA, followed by linolenic, oleic, and palmitic acids [38]. Chokeberry seed oil, previously extracted using SFE-CO_2_, showed a high content of linoleic acid (above 70%), followed by oleic, α-linolenic, and palmitic acids [39].

Moreover, SFE**_EX_** showed a high content of β-carotene and tocopherols, and α-tocopherol was found to be present in the largest amount, followed by β-tocopherol, and γ-tocopherol. According to our data, α-tocopherol was previously reported to be the predominant tocopherol in seed oil from Bulgarian chokeberry, with an average percentage contribution of 70%, followed by β-tocopherol [19]. Fractions of oil extracts obtained during the supercritical extraction on a semi-industrial scale from chokeberry seeds showed a high content of α-tocopherol (in the range of 0.17–1.10 mg/g) [39]. Different values for total carotenoids were reported in *A. melanocarpa* berries, from 0.0077 to 0.0167 mg/g fw [5] and 48.6 mg/kg [11,41]. Among carotenoids, β-carotene has been identified as the most abundant component in berries, and various amounts were reported in the literature, including 16.7 μg/g of whole fruit [41], 46.4 μg/g dw [42], and 4.95–8.87 μg/g [11].

UAM [25,26] with methanol at room temperature (25 °C) for 30 min was applied to obtain a phenol-enriched extract (UAM_M_). Among the extracted polyphenols, flavonoids (mainly flavonols and anthocyanins) were the most abundant class in UAM_M_, followed by phenolic acids, and quercetin-sulfate emerged as the major UAM_M_ component.

Several studies have demonstrated that *A. melanocarpa* is a rich source of polyphenols, mainly phenolic acids and flavonoids, with several health effects [9]. According to our results, flavonols represent one of the most abundant classes of flavonoids, with quercetin derivatives as the most abundant compounds [43,44,45]. However, isorhamnetin and kaempferol derivatives were also previously detected [44,46]. Anthocyanins represented the second largest group of flavonoids (15–30%), being black chokeberry among the anthocyanin-rich berries like blackcurrant and elderberry [43]. The most abundant compounds include cyanidin and pelargonidin derivatives [43,45,47], whereas this is the first study that also identified delphinidin, peonidin, and malvidin derivatives.

*A. melanocarpa* is also characterized by a high amount of hydroxycinnamic derivatives such as caffeic, coumaric, and ferulic derivatives [44], and minor sub-classes of flavonoids such as flavones and flavanols, of which the most representative compounds are certainly apigenin and luteolin, and catechin and epicatechin derivatives, respectively [45,46,47]. Eryodictiol-3-*O*-glucoside is the only flavanone previously identified in *A. melanocarpa*, although in the present study, other compounds belonging to the same sub-class of polyphenols, such as naringenin derivatives (i.e., phloretin and phloridzin), were identified.

Despite being well-known that iminogroups of polyamines such as putrescine, spermine, and spermidine can interact with hydroxycinnamic acids, thus giving rise to mono-, di-, or tris-ubstituted derivatives of spermidine, this is the first study which identified these compounds in *A. melanocarpa* fruit extract. To confirm these results, a tri-p-coumaroylspermidine derivative has been already identified in flowers of plants belonging to the Rosaceae family [48].

Many authors have demonstrated that black chokeberry represents a source of natural antioxidants, and the antioxidant activity of *A. melanocarpa* extracts has been strongly correlated with the content of total phenolics [5,6,7,8,9,11,13,14]. Methanol and aqueous-methanol extracts obtained from black chokeberries showed antioxidant/antiradical activity in DPPH and ABTS assays [11,49]. Therefore, the antioxidant properties of the phenol-enriched extract UAM_M_ were investigated in a biochemical assay of oxidative stress, namely, the solvent-free degradation of cholesterol for 1 h at 140 °C. This in vitro model of lipid oxidation has been amply used to assess the antioxidant properties of natural extracts and pure compounds [29,50,51]. Cholesterol, a lipid of high nutritional value, easily undergoes enzymatic and non-enzymatic oxidation, leading to a wide variety of oxidation products named oxysterols [51,52,53]. Dietary intake of oxysterols has been reported to be a risk for human health [51,52,53]. Solid cholesterol films are very resistant to autoxidation in a dry state. Nevertheless, a sudden increase in oxidation rate occurs at temperatures close to melting point [29,54]. The 7-position on cholesterol seems to be the most reactive with oxygen [53]. During non-enzymatical oxidation of cholesterol, peroxide and hydroxyl groups often form first and then further oxidize into 7-keto [53]. 7-Oxygenated oxysterols, such as 7-OH and 7-keto are the major and more stable oxysterols formed during cholesterol oxidation at 140 °C [29,50,51,54]. 7-Keto is the most common product of a reaction between cholesterol and oxygen radicals [53] and is the most concentrated oxysterol found after dry heating cholesterol films at 140 °C for 1 h [29,50,51,54]. UAM_M_ exerted significant protection of cholesterol from degradation, with an IA_50_ value of 30.6 μg, significantly reducing, in comparison to oxidized samples, the formation of the oxysterols 7β-OH and 7-keto. UAM_M_ protection against cholesterol oxidative degradation was attributable to the scavenging ability of extract components against peroxyl radicals, the main radicals formed in this system [29,51].

The UAM_M_ antioxidant activity in protecting sterol against free-radical attack at 1 h-oxidation was similar to that previously observed, in the same experimental condition, for the water extract (total phenols amounts: 0.52 g of gallic acid equivalents/L of extract) obtained using maceration of the berries (with an IA_50_ value of 39.2 μg) of myrtle (*Myrtus communis*), a plant amply explored for its antioxidant properties [50]. Moreover, the antioxidant effect of UAM_M_ versus the cholesterol oxidative process (1 h) was comparable, in the range of 25–100 μg, to that of strawberry tree honey, which exhibited the highest antioxidant potential among a series of different honeys, strictly related to its high amount of total phenols (972 mg/kg of gallic acid equivalents) [29].

Several studies evidenced the cytotoxic and pro-apoptotic effect of extracts and pure compounds isolated from black chokeberries in various cancer cell lines (human breast, leukemia, glioblastoma, colon, and cervical tumor cells, among others) [7,8,9,13,14,18,42]. UAM_M_ was therefore evaluated for its ability to affect viability in human skin melanoma A375 cells, a cultured cancer cell model extensively used in the prediction of the potential antitumor properties of natural compounds/extracts in cutaneous melanoma [27,28], one of most therapy-resistant types of cancer. UAM_M_ significantly reduced viability in A375 melanoma cells in the range 500–2000 μg/mL after 96 h of incubation.

The UAM_M_ cytotoxic effect was lower than the cytotoxicity previously observed after treatment of A375 cells with the ethanolic extract obtained using maceration from *A. melanocarpa* (Michx.) Elliott dried berries (38.7% of cell viability, versus control, at 250 μg/mL of extract after 72 h-incubation) [7]. The observed differences in the growth inhibitory effect of the two black chokeberry extracts could be ascribable to differences in the polarity of the solvent used for the extraction that affects the type and quantity of extracted phenols/antioxidants [42,55].

UAM_M_ evidenced a significant lower cytotoxicity in normal HaCaT keratinocytes than in melanoma cells, confirming the selective toxic effect of *A. melanocarpa* extracts toward cancer cells [7,14].

## 4. Materials and Methods

### 4.1. Chemicals and Reagents

All the chemicals used in this study were of analytical grade. Standards of fatty acids (FA) (purity ≥98%), cholesterol, 5-cholesten-3β-ol-7-one (7-keto), 5-cholestene-3β,7β-diol (7β-OH), and all solvents used (purity ≥ 99.9%), were purchased from Sigma-Aldrich (Milan, Italy). cis,trans-13-Hydroperoxyoctadecadienoic acid and cis,trans-9-hydroperoxyoctadecadienoic acid (standards of conjugated diene hydroperoxides from linoleic acid) were purchased from Cascade (Cascade Biochem. Ltd., London, UK). Cell culture material was supplied by Invitrogen (Milan, Italy).

### 4.2. Plant Material

Dried fruits of *A. melanocarpa* (Michaux) S. Elliot were supplied by Minardi (Bagnacavallo-Ravenna, Italy; lot number C-300B21-21, plant cultivated in the Republic of North Macedonia). The water content was 10% (*w*/*w*) on a dry basis. Before utilization, the plant material was ground with the Malavasi mill (Bologna, Italy), taking care to avoid overheating. Ground vegetable material was sieved using mechanical sieving, with particles sizes in the range of 250–425 μm. The vegetable matrix was subjected to three different extraction methods (supercritical CO_2_ extraction, maceration, and hydrodistillation).

### 4.3. Apparatus for Supercritical CO_2_ Extraction

SFE-CO_2_ extraction was performed in a laboratory apparatus equipped with a 320 cm^3^ extraction vessel, as previously reported [33]. Extraction was carried out for 4 h in the semi-batch mode, which involved the batch charging of vegetable matter and continuous flow solvent, adopting an experimental arrangement that leaves out the first separator. About 300 g of fruits was charged in each run. Operative conditions were 200 bar and 40 °C in the extraction section and 20 bar and 15 °C in the separator. The CO_2_ flow (Φ) during extraction was 1.2 kg/h. The obtained extract SFE_EX_ was stored at −20 °C before chemical assays.

### 4.4. Ultrasound Assisted Maceration

Solvent extraction was performed using UAM with methanol to obtain the phenolic-rich extract and n-hexane to compare the extraction performances with SFE-CO_2_. Plant materials (10 g) were macerated in 120 mL of methanol or n-hexane. The process was performed in an ultrasonic bath at 40 kHz for 30 min at 25 °C, taking care to avoid overheating. After filtration, methanol (UAM_M_) and n-hexane (UAM_H_) extracts were concentrated using a rotary evaporator under vacuum.

### 4.5. Hydrodistillation

HD was performed for 4 h in a circulatory Clevenger-type apparatus up to the exhaustion of the oil (HD_EX_) contained in the matrix, according to the procedure described in the European Pharmacopoeia [56].

### 4.6. Essential Oil Analysis

Analyses of HD_EX_ were carried out using gas chromatography/mass spectrometry (GC-MS) using a gas chromatograph (Agilent 6890N) equipped with a 30 m × 0.25 mm i.d. with 0.25 µm stationary film thickness HP-5ms capillary column (Agilent J&W) coupled with a mass selective detector having an electron ionization device, EI, and a quadrupole analyzer (Agilent 5973). The following temperature program was used: from 60 °C to 246 °C at a rate of 3 °C min^−1^, and then held at 246 °C for 20 min (total analysis time 82 min). Other operating conditions were the following: carrier gas helium (purity ≥ 99.9999%, Air Liquide Italy); flow rate, 1.0 mL/min; injector temperature, 250 °C; and detector temperature, 300 °C. Injection of 1 μL of diluted sample (1:100 in n-hexane, *w*/*w*) was performed with 1:20 split ratio using an autosampler (Agilent, Model 7683B).

The MS conditions were as follows: MS transfer line temperature 240 °C; EI ion source temperature, 200 °C with ionization energy of 70 eV; quadrupole temperature 150 °C; and scan rate, 3.2 scan s^−1^ at *m*/*z* scan range, (30 to 480). The software MSD ChemStation (Agilent, rev. E.01.00.237) was used to handle and process chromatograms and mass spectra. Compounds were identified using a comparison of their mass spectra with those of NIST02 the library data [31] of the GC/MS system and Adams libraries spectra [30]. The results were further confirmed using a comparison with the compound’s elution order with their retention indices on semi-polar phases reported in the literature [30]. Retention indices of the components were determined relative to the retention times of a series of n-alkanes (two standard mixes C_8_–C_20_ and C_21_–C_40_) with linear interpolation [57]. The percentage of individual components was calculated based on GC peak areas without FID response factor correction.

### 4.7. Polyphenols Analysis Using LC-ESI-MS

The phytochemical profile of UAM_M_ was investigated using LC-ESI-MS analysis according to Smeriglio et al. [58]. Chromatographic elution was carried out using a Luna Omega PS C18 column (150 mm x 2.1 mm, 5 µm; Phenomenex, Torrance, CA, USA) at 25 °C by using a mobile phase consisting of 0.1% formic acid (Solvent A) and acetonitrile (Solvent B) according to the following program: 0–3 min, 0% B; 3–9 min, 3% B; 9–24 min, 12% B; 24–30 min, 20% B; 30–33 min, 20% B; 33–43 min, 30% B; 43–63 min, 50% B; 63–66 min, 50% B; 66–76 min, 60% B; 76–81 min, 60% B; and 81–86 min, 0% B and equilibrated 4 min. The injection volume was 5 µL. The experimental parameters of the mass spectrometer (ion trap, model 6320, Agilent Technologies, Santa Clara, CA, USA), operating in negative and positive ionization mode, were set as follows: 3.5 kV capillary voltage, 40 psi nebulizer (N_2_) pressure, 350 °C drying gas temperature, and 9 L/min drying gas flow. Mass spectra were acquired in full-scan mode (90–1000 *m*/*z*). The identification was carried out by comparing the retention times and mass spectra of each analyte with those of commercially available reference standards (HPLC-grade, ≥ 95%, see Table 2 and relative footnote for details), as well as with the literature data and open source mass spectra databases.

### 4.8. Saponification of SFE_EX_ and UAM_M_ for Fatty Acid Preparation

SFE_EX_ and UAM_M_ obtained from *A. melanocarpa* were subjected to mild saponification as follows: 50 μL of Desferal solution (25 mg/mL of H_2_O), 500 μL of a water solution of ascorbic acid (25% *w*/*v*), and 250 μL of 10 N KOH were added to aliquots (1.5 mg) of SFE**_EX_** and UAM_H_ in ethanol solution [33]. The mixtures were left in the dark at room temperature for 14 h. Then, after the addition of n-hexane (5 mL) and H_2_O (3.5 mL), samples were acidified to pH 3–4 with 37% HCl, and then were centrifuged for 1 h at 900× *g*. The hexane phase (saponifiable fraction), containing free FA and conjugated diene fatty acid hydroperoxides (HP), was collected, the solvent was evaporated under vacuum, and the dried residues was dissolved in acetonitrile with 0.14% acetic acid (*v*/*v*) [33]. Aliquots were then analyzed using liquid chromatography for FA composition [33].

### 4.9. Fatty Acid Analysis

Analyses were carried out with an Agilent Technologies 1100 HPLC equipped with a DAD and an Infinity 1260 ELSD (Agilent Technologies, Palo Alto, CA, USA). Analyses of FA (unsaturated were detected at 200 nm, saturated with ELSD) and HP (detected at 234 nm) were performed with an XDB-C18 Eclipse column equipped with a Zorbax XDB-C_18_ Eclipse guard column (Agilent Technologies), with a mobile phase of acetonitrile/water/acetic acid (75/25/0.12, *v*/*v*/*v*), at a flow rate of 2.3 mL/min, as previously described [33]. An Agilent OpenLAB Chromatography data system was used for the recording and integration of the chromatogram data. Calibration curves were constructed with standard compounds (correlation coefficients > 0.995), and were found to be linear and quadratic for DAD and ELSD detectors, respectively.

### 4.10. Carotenoid and Tocopherols Analysis of SFE_EX_ Using HPLC-DAD and HPLC-FLU

The quali-quantitative analysis of carotenoids was carried out with HPLC-DAD analysis using a LiChrosorb SI-60 analytical column (250 × 4.6 mm, 5 µm, Supelco Inc., Bellefonte, PA, USA) at 22 °C. An isocratic elution was carried out by using hexane/acetone (82:18, *v*/*v*) as the mobile phase. The injection volume was 10 µL. The UV–Vis spectra were recorded ranging from 190 to 600 nm. The acquisition at 474 nm was used to identify and quantify the β-carotene content by using the external standard calibration curve of the reference standard (HPLC-grade, ≥95%, C4582 Merck KGaA, Darmstadt, Germany). The results were expressed as mg/100 g of SFE**_EX_**, and represent the mean ± standard deviation of three independent analyses in triplicate (*n* = 3).

The quali-quantitative analysis of tocopherols was carried out, as previously reported [59], with HPLC-FLU analysis using the above analytical column at 25 °C. N-heptane/tetrahydrofurane (96.15:3.85, *v*/*v*) with a flow rate of 1.0 mL/min was used as the mobile phase. Fluorescence detection (λ_ex_ 295 nm, λ_em_ 330 nm) was used to identify and quantify the α, β, γ, and δ tocopherol content by using external standard calibration curves of the four reference standards (HPLC-grade ≥ 90% 47783, V-082, T1782, and 47784, respectively, from Merck KGaA, Darmstadt, Germany). The results were expressed as mg of α-δ tocopherol/100 g of SFE_EX_.

### 4.11. Cholesterol Oxidation Assay

The protective effect of aliquots of UAM_M_ was evaluated during the cholesterol oxidation in a dry state, as previously reported [29,51]. Aliquots of 0.5 mL of cholesterol solution (2 mg/mL of methanol), in the absence (oxidized controls) and in the presence of different aliquots (2.5–100 μg) of UAM_M_ (from a solution 0.5 mg/mL in methanol), were dried in a round-bottom test tube under vacuum and then incubated in a bath at 140 °C for 1 h under artificial light exposure; controls (Ctrl, non-oxidized cholesterol) were kept at 0 °C in the dark. HPLC-DAD analyses of cholesterol, 7-ketocholesterol (7-keto), and 7β-hydroxycholesterol (7β-OH) were carried out as previously described [29,51].

### 4.12. Cell Culture

Human malignant A375 melanoma cells were purchased from the American Type Culture Collection (ATCC, Rockville, MD, USA). HaCaT cell line (human keratinocyte cells) was obtained from CLS-Cell Line Services (Eppelheim, Germany). Both cell lines were grown in Dulbecco’s modified Eagle’s medium (DMEM) with high glucose, supplemented with fetal calf serum (FCS) (10% *v*/*v*) and 2 mM L-glutamine, penicillin (100 units/mL)–streptomycin (100 μg/mL), at 37 °C in a 5% CO_2_ incubator. Subcultures of A375 and HaCaT cells were grown in T-75 culture flasks and passaged with a trypsin-EDTA solution.

### 4.13. Evaluation of Cytotoxic Effect Using MTT Viability Assay

UAM_M_ cytotoxic effect was measured in A375 melanoma cells and HaCaT keratinocytes using the MTT viability colorimetric assay [54,60]. A375 cancer cells (at a density of 3 × 10^4^ cells/mL) and HaCaT keratinocytes (at a density of 7 × 10^4^ cells/mL) were seeded in 96-well plates in a complete culture medium (100 μL). After 48 h-incubation, treated cells were incubated (for 96 h) in a fresh medium with various concentrations (10–2000 μg/mL) of UAM_M_ (from a solution of 20 mg/mL in ultrapure water). Then, control (non-treated) cells and UAM_M_-treated cells were subjected to the MTT viability test as previously reported [60]. The auto micro-plate reader (Infinite 200, Tecan, Grödig, Austria) was used to measure color development at the wavelength of 570 nm. The absorbance measured in each well was proportional to the number of viable cells, and results were expressed as a percentage of cell viability compared to control cells.

### 4.14. Statistical Analyses

Results were expressed as mean and standard deviation. The evaluation of statistical differences was performed with Graph Pad INSTAT 3.3 software (GraphPad Software, San Diego, CA, USA). Multiple comparisons of the group means were assessed using one-way analysis of variance (one-way ANOVA) followed by the Bonferroni Multiple Comparisons Test. Student’s unpaired *t*-test with Welch’s correction was used to compare the means of the two groups. The minimal level of significance was *p* < 0.05.

## 5. Conclusions

The chemical composition, nutritional, and biological properties of extracts obtained from *A. melanocarpa* berries using different extraction methods/solvents were studied. Hydrodistillation of dried black chokeberries led to an essential oil (HD_EX_) characterized by a high content of oxygenated sesquiterpenes, and italicene epoxide was identified as its major component. The application of SFE-CO_2_ extraction, an environmentally friendly method, furnished a berry-fixed oil (SFE_EX_) rich in the essential FA 18:2, characterized by high levels of the bioactive compounds β-carotene and α-tocopherol. A mild- and short-time method (ultrasound-assisted maceration for 30 min) gave a phenol-enriched extract (UAM_M_) rich in flavonols, anthocyanins, and phenolic acids. UAM_M_ exhibited the ability to protect cholesterol, a structural component of cell membranes and food lipid compound, against oxidative degradation, and reduced viability in A375 melanoma cells, a model of cutaneous melanoma.

The results of our research extend the knowledge of nutritional and biological properties of *A. melanocarpa* berries, providing useful information on various methods to obtain specific extracts (essential oil, fixed oil, and a mixture of phenolic compounds) of identified composition for potential applications in the food, cosmetic, and pharmaceutical industries. Further studies are needed to assess the biological effect of isolated extracts in various normal and cancer cells.

## Figures and Tables

**Table 1:** Tentative identification of volatile profile of *A. melanocarpa* fruit obtained using hydrodistillation (HD). Results were measured using GC-MS analysis, and are expressed as mean area percentage (%).

Number	t_R_ (min)	RI_EXP_ ^1^	RI_LIT_	Compound	% Area	Formula	Class
1	6.1647	960	952	Benzaldehyde	1.6	C_7_H_6_O	Other
2	6.9502	989	988	Myrcene	0.2	C_10_H_16_	HM
3	8.2888	1028	1024	Limonene	9.7	C_10_H_16_	HM
4	9.1624	1052		n.i.	0.2		
5	14.0117	1174	1167	Menthol	0.5	C_10_H_20_O	OM
6	14.2201	1179	1178	Naphthalene	0.7	C_10_H_8_	Other
7	16.7609	1238	1239	Carvone	0.7	C_10_H_14_O	OM
8	18.3079	1274	1270 *	Vitispirane *	0.8	C_13_H_20_O	Other
9	18.5964	1281	1282	(E)-Anethole	6.1	C_10_H_12_O	OM
10	19.1495	1294	1298	Carvacrol	0.5	C_10_H_14_O	OM
11	24.1991	1413		n.i.	0.4		
12	24.2873	1415	1421	Linalool butanoate	0.3	C_14_H_24_O_2_	OM
13	25.4014	1442	1434	Neryl acetone	1.4	C_13_H_22_O	OM
14	26.3392	1465	1475	trans-Cadina-1(6),4-diene	0.3	C_15_H_24_	HS
15	26.5957	1471		n.i.	2.0		
16	26.74	1475	1479	Ar-curcumene	0.7	C_15_H_22_	HS
17	27.1247	1484		n.i.	0.3		
18	28.2068	1511	1511	δ-Amorphene	2.9	C_15_H_24_	HS
19	28.3671	1515	1513	γ-Cadinene	1.3	C_15_H_24_	HS
20	28.7358	1524	1533	trans-Cadina-1,4-diene	0.3	C_15_H_24_	HS
21	29.2488	1538	1547	Italicene epoxide	17.2	C_15_H_24_O	OS
22	30.403	1567	1565	Dodecanoic acid	9.7	C_12_H_24_O_2_	Other
23	32.3908	1619	1618	1,10-di-epi-Cubenol	1.3	C_15_H_26_O	OS
24	32.9519	1634		n.i.	1.2		
25	33.1041	1638	1644	α-Muurolol	0.6	C_15_H_26_O	OS
26	33.7373	1655		n.i.	0.9		
27	33.8896	1659		n.i.	1.1		
28	34.7633	1682	1679	Khusinol	10.5	C_15_H_24_O	OS
29	36.1339	1720	1724	(Z)-Nuciferol	0.7	C_15_H_22_O	OS
30	37.4725	1757		n.i.	1.5		
31	40.1897	1835		n.i.	0.4		
32	40.2778	1838		n.i.	0.4		
33	40.6305	1849		n.i.	2.4		
34	40.871	1856		n.i.	0.9		
35	42.4099	1901	1889	(5Z,9E)-Farnesyl acetone	0.6	C_18_H_30_O	OS
36	43.011	1920	1921	methyl Hexadecanoate	0.5	C_17_H_34_O_2_	Other
37	43.7485	1942		n.i.	0.4		
38	44.3336	1960	1959	Hexadecanoic acid	12.4	C_16_H_32_O_2_	Other
39	49.4153	2124	2132	Linoleic acid	3.1	C_18_H_32_O_2_	Other
40	49.5435	2128	2141	Oleic acid	1.0	C_18_H_34_O_2_	Other
41	66.6001	2750		n.i.	1.0		
42	71.7619	2891	2900	Nonacosane	1.1	C_29_H_60_	Other
Total identified					86.7		
Hydrocarbon monoterpenes (HM)					9.9		
Oxygenated monoterpenes (OM)					9.5		
Hydrocarbon sesquiterpenes (HS)					5.5		
Oxygenated sesquiterpenes (OS)					30.9		
Other compounds (Other)					30.9		

^1^ RI_EXP_, retention index determined on a HP-5ms fused silica column relative to a series of n-alkanes. Compounds were identified by comparing their retention indices (RI_EXP_) and mass spectra (MS) with those reported in Adams (RI_LIT_) [30] and NIST02 [31] libraries. n.i.= not identified. * Identified using the literature comparison [32]. RI_EXP_ is calculated according to the following formula: RI_EXP_ = $\left[{\left(t_{R}\right)}_{i}-{\left(t_{R}\right)}_{z}/{\left(t_{R}\right)}_{z+1}-{\left(t_{R}\right)}_{z}\right]\times 100+100\times z$, where z and z + 1 are the number of carbon atoms of the eluting alkanes before and after component i, respectively [30].

**Table 2:** Tentative identification of phytochemical profile of *A. melanocarpa* fruit ultrasound-assisted methanol extract (UAM_M_) by LC-ESI-MS analysis. Results were expressed as mean area percentage (%) ± standard deviation (*n* = 3) of each compound with respect to the total area of polyphenols identified.

Compound	RT * (min)	[M − H]^−^/[M + H]^+^ *m*/*z*	Area (%)
Dihydrocaffeic acid ^a^	0.9	181/	0.89 ± 0.04
(−)-Epigallocatechin-glucuronide	2.5	/483	3.32 ± 0.15
Methyl-epigallocatechin	2.9	/321	5.68 ± 0.22
Galloyl glucose	3.6	/333	2.28 ± 0.12
Quercetin-sulfate	4.2	/383	15.58 ± 0.56
Myricetin 3-arabinoside	9.4	/451	1.11 ± 0.03
Eriodictyol-7-*O*-glucoside ^a^	10.2	/451	1.95 ± 0.04
Chlorogenic acid ^a^	14.1	353/	0.16 ± 0.01
Quercetin-O-arabinoside	18.0	/435	1.83 ± 0.04
Feruloyl glucose	20.8	355/	0.31 ± 0.01
Cyanidin-3-*O*-glucoside ^a^	22.7	/449	1.39 ± 0.03
Hexahydroxydiphenoyl-glucose	24.1	481/	0.04 ± 0.00
Pelargonodin-rutinoside-glicoside	25.8	/742	0.91 ± 0.02
Myricetin-3-*O*-galactoside ^a^	27.4	/481	1.80 ± 0.08
Ligstroside	29.1	/525	2.19 ± 0.14
Phloretin-xylosyl-glucoside	30.4	/569	2.16 ± 0.05
Delphinidin-3-*O*-glucoside ^a^	30.8	464/	0.08 ± 0.00
Quercetin-3-sambubioside	31.7	595/	0.05 ± 0.00
Luteolin 7-*O*-apiosyl-gucoside	32.2	/581	1.68 ± 0.05
Rutin ^a^	32.7	609/	0.15 ± 0.01
Delphinidin-3-*O*-galactoside ^a^	33.0	464/	0.16 ± 0.01
Isorhamnetin-3-*O*-glucoside 7-*O*-rhamnoside	33.8	/625	1.61 ± 0.06
Quercetin-pentoside-deoxydihexoside	34.6	/743	1.22 ± 0.08
Quercetin-*O*-rhamnose-*O*-glucose	34.8	609/	0.04 ± 0.00
Quercetin 3-sophoroside	35.6	/625	1.55 ± 0.02
(-)-Epigallocatechin-gallate ^a^	36.2	457/	0.03 ± 0.00
1-Sinapoyl-2-feruloylgentiobiose	36.6	723/	0.04 ± 0.00
Phloridzin	37.3	/437	1.69 ± 0.07
Pectolinarin	37.8	/623	0.00 ± 0.00
Resveratrol-3-*O*-beta-D-glucopyranoside (Piceid) ^a^	38.2	/391	1.31 ± 0.02
Pelargonidin 3-(6″-acetyl) glucoside	38.6	353/	0.03 ± 0.00
Pelargonidin-*O*-malonyl-glucoside	39.9	518/	0.05 ± 0.00
Myricetin ^a^	40.9	/319	1.89 ± 0.04
Methoxybenzoic acid rhamnoside	41.6	297/	1.69 ± 0.05
Procyanidin dimer	42.0	577/	0.04 ± 0.00
Resveratrol trimer	42.8	/679	1.90 ± 0.02
Cichoric acid ^a^	43.5	474/	2.86 ± 0.12
Procyanidin dimer	44.4	577/	0.04 ± 0.00
Phloretin ^a^	45.6	/275	4.09 ± 0.18
Procyanidin dimer	46.8	577/	0.04 ± 0.00
6-Geranylnaringenin	47.1	407/	0.13 ± 0.01
5-Pentacosenylresorcinol	47.9	457/	0.03 ± 0.00
5-Pentacosylresorcinol	51.8	459/	0.06 ± 0.00
Caffeoyl di-coumaroyl spermidine	70.7	/600	3.95 ± 0.12
Coumaroyl di-feruoyl spermidine	73.1	/644	3.70 ± 0.17
di-Caffeoyl coumaroyl spermidine	74.4	/616	7.60 ± 0.14
Peonidin-*O*-acetyl-glicoside	56.3	/506	2.09 ± 0.07
Caffeoyl glucose	57.0	/343	0.00 ± 0.00
Kaempferol-3-*O*-rhamnosyl-galactoside	58.1	593/	0.00 ± 0.00
Apigenin 6-C-α-L-arabinosyl-8-C-β-L-arabinoside	60.7	533/	1.23 ± 0.02
Luteolin 7-*O*-(2-apiosyl-6-acetyl) glucoside	62.0	/623	1.63 ± 0.05
Digalloyl quinic acid	62.8	495/	0.35 ± 0.01
5-Carboxypyranocyanidin-3-pentoside	64.1	/488	2.94 ± 0.08
Quercetin hexose glucuronide	64.4	639/	0.06 ± 0.00
Galloyl-hexahydroxydiphenoylglucose	65.4	633/	0.06 ± 0.00
Peonidin 3-*O*-sophoroside	65.8	/626	3.05 ± 0.08
Malvidin 3-*O*-(6-*O*-p-coumaroyl) glucoside-4-vinylphenol adduct (pigment B)	79.0	/756	9.28 ± 0.18

* RT, Retention time; ^a^ confirmed by injection of reference standards.

## Data Availability

The datasets generated and analyzed during the current study are available from the corresponding author on reasonable request.

## References

[1] Najmi A., Javed S.A., Al Bratty M., Alhazmi H.A. (2022). Modern approaches in the discovery and development of plant-based natural products and their analogues as potential therapeutic agents. Molecules.

[2] Nurzyńska-Wierdak R. (2023). Phenolic compounds from new natural sources—Plant genotype and ontogenetic variation. Molecules.

[3] Rosa A., Rescigno A., Piras A., Atzeri A., Scano P., Porcedda S., Zucca P., Dessì M.A. (2012). Chemical composition and effect on intestinal Caco-2 cell viability and lipid profile of fixed oil from *Cynomorium coccineum* L. Food Chem. Toxicol..

[4] Berwal M.K., Ram C., Gurjar P.S., Gora J.S., Kumar R., Verma A.K., Singh D., Basile B., Rouphael Y., Kumar P. (2022). The Bioactive compounds and fatty acid profile of bitter apple seed oil obtained in hot, arid environments. Horticulturae.

[5] Borowska S., Brzóska M.M. (2016). Chokeberries (*Aronia melanocarpa*) and their products as a possible means for the prevention and treatment of noncommunicable diseases and unfavorable health effects due to exposure to xenobiotics. Compr. Rev. Food Sci. Food Saf..

[6] Denev P., Kratchanova M., Petrova I., Klisurova D., Georgiev Y., Ognyanov M., Yanakieva I. (2018). Black chokeberry (*Aronia melanocarpa* (Michx.) Elliot fruits and functional drinks differ significantly in their chemical composition and antioxidant activity. J. Chem..

[7] Buda V., Brezoiu A.-M., Berger D., Pavel I.Z., Muntean D., Minda D., Dehelean C.A., Soica C., Diaconeasa Z., Folescu R. (2020). Biological evaluation of black chokeberry extract free and embedded in two mesoporous silica-type matrices. Pharmaceutics.

[8] Jurikova T., Mlcek J., Skrovankova S., Sumczynski D., Sochor J., Hlavacova I., Snopek L., Orsavova J. (2017). Fruits of black chokeberry *Aronia melanocarpa* in the prevention of chronic diseases. Molecules.

[9] Jurendić T., Ščetar M. (2021). *Aronia melanocarpa* products and by-products for health and nutrition: A review. Antioxidants.

[10] Wójtowicz A., Combrzyński M., Biernacka B., Różyło R., Bąkowski M., Wojtunik-Kulesza K., Mołdoch J., Kowalska I. (2023). Fresh chokeberry (*Aronia melanocarpa*) fruits as valuable additive in extruded snack pellets: Selected nutritional and physiochemical properties. Plants.

[11] Sidor A., Gramza-Michałowska A. (2019). Black chokeberry *Aronia melanocarpa* L.—A qualitative composition, phenolic profile and antioxidant potential. Molecules.

[12] Lee H.-Y., Suh K.S., Kim Y.I., Jang B.-K., Kim B.-H., Yim S.-V. (2021). Bioactive Fraction of *Aronia melanocarpa* fruit inhibits adipogenic differentiation of cultured 3T3-L1 cells. Appl. Sci..

[13] Kulling S.E., Rawel H.M. (2008). Chokeberry (*Aronia melanocarpa*)—A review on the characteristic components and potential health effects. Planta Med..

[14] Ren Y., Frank T., Meyer G., Lei J., Grebenc J.R., Slaughter R., Gao Y.G., Kinghorn A.D. (2022). Potential benefits of black chokeberry (*Aronia melanocarpa*) fruits and their constituents in improving human health. Molecules.

[15] Martin D.A., Taheri R., Brand M.H., Draghi A., Sylvester F.A., Bolling B.W. (2014). Anti-inflammatory activity of aronia berry extracts in murine splenocytes. J. Funct. Foods.

[16] Mikami N., Hosotani Y., Saso T., Ohta T., Miyashita K., Hosokawa M. (2020). Black chokeberry (*Aronia melanocarpa*) juice residue and its ethanol extract decrease serum lipid levels in high-fat diet-fed C57BL/6J mice. Int. J. Funct. Nutr..

[17] Ćujić N., Šavikin K., Jankovic’ T., Pljevljakušic´ D., Zdunic´ G., Ibric´ S. (2016). Optimization of polyphenols extraction from dried chokeberry using maceration as traditional technique. Food Chem..

[18] Šavikin K., Zduni´c G., Jankovi´c T., Goevac D., Stanojkovi´c T., Pljevljakuši´c D. (2014). Berry fruit teas: Phenolic composition and cytotoxic activity. Food Res. Int..

[19] Zlatanov M.D. (1999). Lipid composition of Bulgarian chokeberry, black currant and rose hip seed oils. J. Sci. Food Agric..

[20] Hirvi T., Honkanen E. (1985). Analysis of the volatile constituents of black chokeberry (*Aronia melanocarpa* Ell.). J. Sci. Food Agric..

[21] Kraujalyte V., Leitner E., Venskutonis P.R. (2013). Characterization of *Aronia melanocarpa* volatiles by head-space-solid-phase microextraction (HS-SPME) simultaneous distillation/extraction (SDE) and gas-chromatography-olfactometry (GC-O). J. Agric. Food Chem..

[22] Piras A., Piras C., Porcedda S., Rosa A. (2021). Comparative evaluation of the composition of vegetable essential and fixed oils obtained by supercritical extraction and conventional techniques: A chemometric approach. Int. J. Food Sci. Technol..

[23] Braga M.E.M., Gaspar M.C., de Sousa H.C. (2023). Supercritical fluid technology for agrifood materials processing. Curr. Opin. Food Sci..

[24] Satriana S., Supardan M.D., Arpi N., Mustapha A.W. (2019). Development of methods used in the extraction of avocado oil. Eur. J. Lipid Sci. Technol..

[25] Ramić M., Vidović S., Zeković Z., Vladić J., Cvejin A., Pavlić B. (2015). Modeling and optimization of ultrasound-assisted extraction of polyphenolic compounds from *Aronia melanocarpa* by-products from filter-tea factory. Ultrason. Sonochem..

[26] Peng S., Zhu M., Li S., Ma X., Hu F. (2023). Ultrasound-assisted extraction of polyphenols from Chinese propolis. Front. Sustain. Food Syst..

[27] Kyriakou S., Tragkola V., Plioukas M., Anestopoulos I., Chatzopoulou P.S., Sarrou E., Trafalis D.T., Deligiorgi M.V., Franco R., Pappa A. (2021). Chemical and biological characterization of the anticancer potency of *Salvia fruticosa* in a model of human malignant melanoma. Plants.

[28] Chen J., Huang C., Liu F., Xu Z., Li L., Huang Z., Zhang H. (2019). Methylwogonin exerts anticancer effects in A375 human malignant melanoma cells through apoptosis induction, DNA damage, cell invasion inhibition and downregulation of the mTOR/PI3K/Akt signalling pathway. Arch. Med. Sci..

[29] Rosa A., Tuberoso C.I.G., Atzeri A., Melis M.P., Bifulco E., Dessì M.A. (2011). Antioxidant profile of strawberry tree honey and its marker homogentisic acid in several models of oxidative stress. Food Chem..

[30] Adams R.P. (2007). Identification of Essential Oil Components by Gas Chromatography/Mass Spectroscopy.

[31] NIST/EPA/NIH (2005). Mass Spectral Library.

[32] Pino J.A., Quijano C.E. (2012). Study of the volatile compounds from plum (*Prunus domestica* L. cv. Horvin) and estimation of their contribution to the fruit aroma. Food Sci. Technol..

[33] Rosa A., Maxia A., Putzu D., Atzeri A., Era B., Fais A., Sanna C., Piras A. (2017). Chemical composition of *Lycium europaeum* fruit oil obtained by supercritical CO_2_ extraction and evaluation of its antioxidant activity, cytotoxicity and cell absorption. Food Chem..

[34] Ali S., Ekbbal R., Salar S., Yasheshwar, Ali S.A., Jaiswal A.K., Singh M., Yadav D.K., Kumar S. (2023). Gaurav Quality standards and pharmacological interventions of natural oils: Current scenario and future perspectives. ACS Omega.

[35] Butorova L., Vitova E., Polovka M. (2016). Comparison of volatiles identified in *Aronia melanocarpa* and *Amelanchier alnifolia* using solid-phase microextraction coupled to gas chromatography-mass spectrometry. J. Food Nutr. Res..

[36] Romani A., Vignolini P., Ieri F., Heimler D. (2016). Polyphenols and volatile compounds in commercial chokeberry (*Aronia melanocarpa*) products. Nat. Prod. Commun..

[37] Mastelić J., Politeo O., Jerković I. (2008). Contribution to the analysis of the essential oil of *Helichrysum italicum* (Roth) G. Don. Determination of ester bonded acids and phenols. Molecules.

[38] Pieszka M., Gogol P., Pietras M., Pieszka M. (2015). Valuable Components of Dried Pomaces of Chokeberry, Black Currant, Strawberry, Apple and Carrot as a Source of Natural Antioxidants and Nutraceuticals in the Animal Diet. Ann. Anim. Sci..

[39] Milala J., Grzelak-Błaszczyk K., Sójka M., Kosmala M., Dobrzyńska-Inger A., Rój E. (2018). Changes of bioactive components in berry seed oils during supercritical CO_2_ extraction. J. Food Process Pres..

[40] Kim O.Y., Song J. (2024). Important roles of linoleic acid and α-linolenic acid in regulating cognitive impairment and neuropsychiatric issues in metabolic-related dementia. Life Sci..

[41] Razungles A., Oszmianski J., Sapis J.C. (1989). Determination of carotenoids in fruits of Rosa sp. (*Rosa canina* and *Rosa rugosa*) and of chokeberry (*Aronia melanocarpa*). J. Food Sci..

[42] Olsson M.E., Gustavsson K.E., Andersson S., Nilsson Å., Duan R.D. (2004). Inhibition of cancer cell proliferation in vitro by fruit and berry extracts and correlations with antioxidant levels. J. Agric. Food Chem..

[43] Rodríguez-Werner M., Winterhalter P., Esatbeyoglu T. (2019). Phenolic composition, radical scavenging activity and an approach for authentication of *Aronia melanocarpa* berries, juice, and pomace. J. Food Sci..

[44] Tasinov O., Dincheva I., Badjakov I., Grupcheva C., Galunska B. (2022). Comparative phytochemical analysis of *Aronia melanocarpa* L. fruit juices on Bulgarian market. Plants.

[45] Gerasimov M.A., Perova I.B., Eller K.I., Akimov M.Y., Sukhanova A.M., Rodionova G.M., Ramenskaya G.V. (2023). Investigation of polyphenolic compounds in different varieties of black chokeberry *Aronia melanocarpa*. Molecules.

[46] Lee J.E., Kim G.S., Park S., Kim Y.H., Kim M.B., Lee W.S., Jeong S.W., Lee S.J., Jin J.S., Shin S.C. (2014). Determination of chokeberry (*Aronia melanocarpa*) polyphenol components using liquid chromatography-tandem mass spectrometry: Overall contribution to antioxidant activity. Food Chem..

[47] Bataraga A., Valkovska V. (2020). Phytochemical profile of Chokeberry (*Aronia melanocarpa*). Key Engineering Materials.

[48] Edreva A.M., Velikova V.B., Tsonev T.D. (2007). Phenylamides in plants. Russ. J. Plant Physiol..

[49] Kaloudi T., Tsimogiannis D., Oreopoulou V. (2022). *Aronia melanocarpa*: Identification and exploitation of its phenolic components. Molecules.

[50] Tuberoso C.I.G., Rosa A., Bifulco E., Melis M.P., Atzeri A., Pirisi F.M., Dessì M.A. (2010). Chemical composition and antioxidant activities of *Myrtus communis* L. berries extracts. Food Chem..

[51] Rosa A., Atzeri A., Deiana M., Melis M.P., Incani A., Minassi A., Cabboi B., Appendino G. (2014). Prenylation preserves antioxidant properties and effect on cell viability of the natural dietary phenol curcumin. Food Res. Int..

[52] Canzoneri F., Leoni V., Rosso G., Risso D., Menta R., Poli G. (2022). Oxysterols as reliable markers of quality and safety in cholesterol containing food ingredients and products. Front. Nutr..

[53] Anderson A., Campo A., Fulton E., Corwin A., Jerome W.G., O’Connor M.S. (2020). 7-Ketocholesterol in disease and aging. Redox Biol..

[54] Rosa A., Atzeri A., Nieddu M., Appendino G. (2017). New insights into the antioxidant activity and cytotoxicity of arzanol and effect of methylation on its biological properties. Chem. Phys. Lipids.

[55] Petreska Stanoeva J., Balshikevska E., Stefova M., Tusevski O., Simic S.G. (2020). Comparison of the effect of acids in solvent mixtures for extraction of phenolic compounds from *Aronia melanocarpa*. Nat. Prod. Commun..

[56] (1997). European Pharmacopoeia.

[57] Van Den Dool H., Kratz P.D. (1963). A generalization of the retention index system including linear temperature programmed gas-liquid chromatography. J. Chromatogr..

[58] Smeriglio A., Ingegneri M., Germanò M.P., Miori L., Battistini G., Betuzzi F., Malaspina P., Trombetta D., Cornara L. (2024). Pharmacognostic evaluation of *Monarda didyma* L. growing in Trentino (Northern Italy) for cosmeceutical applications. Plants.

[59] Smeriglio A., Toscano G., Denaro M., De Francesco C., Agozzino S., Trombetta D. (2019). Nitrogen headspace improves the extra virgin olive oil shelf-life, preserving its functional properties. Antioxidants.

[60] Petretto G.L., Vacca G., Addis R., Pintore G., Nieddu M., Piras F., Sogos V., Fancello F., Zara S., Rosa A. (2023). Waste *Citrus limon* leaves as source of essential oil rich in limonene and citral: Chemical characterization, antimicrobial and antioxidant properties, and effects on cancer cell viability. Antioxidants.

